# Current Knowledge on UTUC Epidemiology in Poland Compared to Europe

**DOI:** 10.3390/cancers18010126

**Published:** 2025-12-30

**Authors:** Iwona Wnętrzak, Urszula Wojciechowska, Jakub Dobruch, Mateusz Czajkowski, Roman Sosnowski, Wojciech Krajewski, Marcin Matuszewski, Piotr Marczyński, Joanna A. Didkowska

**Affiliations:** 1 Department of Urology, Centre of Postgraduate Medical Education, Independent Public Hospital of Professor W. Orlowski, 00-416 Warsaw, Poland; 2 Polish National Cancer Registry, Maria Sklodowska-Curie National Research Institute of Oncology, 02-781 Warsaw, Poland; 3 Department of Urology, Medical University of Gdańsk, Mariana Smoluchowskiego 17 Street, 80-214 Gdańsk, Poland; mateusz.czajkowski@gumed.edu.pl (M.C.);; 4 Department of Urology and Oncological Urology, MSWiA Hospital, Warmian-Masurian Cancer Center, 10-228 Olsztyn, Poland; 5 Department of Minimally Invasive and Robotic Urology, University Center of Excellence in Urology, Wrocław Medical University, 50-367 Wrocław, Poland; 6 Mazovia Hospital Warsaw, Department of Urology, 02-797 Warsaw, Poland; 7 Department of Epidemiology and Cancer Prevention, Maria Sklodowska-Curie National Research Institute of Oncology, 02-781 Warsaw, Poland

**Keywords:** epidemiology, incidence, mortality, renal pelvis cancer, trends, sex, upper tract urothelial carcinoma (UTUC), ureteral cancer

## Abstract

**Background:** Upper tract urothelial carcinoma (UTUC) is a rare malignancy with limited epidemiological data from Central and Eastern Europe. This study aimed to evaluate long-term trends in UTUC incidence, mortality, and survival in Poland and to compare them with other European countries. **Methods:** Incidence and mortality data were acquired from Polish National Cancer Registry, Statistics Poland and European Cancer Information System. Age-standardized incidence and mortality rates were calculated using the European Standard Population (ESP 2013). Temporal trends were assessed using Joinpoint regression, and five-year relative survival was estimated with the Pohar–Perme method. **Results:** Our study demonstrated significantly higher incidence and mortality rates of UTUC across Europe compared to the initial observation period, consistent with the data from Poland. UTUC incidence increased in Poland between 2000 and 2017 (average annual percent change [AAPC] 5.6% in women and 9.9% in men), followed by a decline among women after 2017 (AAPC −1.3%). Mortality initially rose in both sexes but decreased in recent years, particularly in men (AAPC −5.47%). Renal pelvis cancer showed higher incidence and mortality than ureteral cancer. Compared with other European countries, Poland exhibited a smaller gap between incidence and mortality. Five-year relative survival improved over time, reaching 55–56% for renal pelvis cancer and up to 62% for ureteral cancer. **Conclusions:** UTUC incidence and mortality are rising in Europe, but Poland displays a distinctive epidemiological pattern. Strengthening national cancer registries and implementing centralized care protocols may improve disease management and reduce mortality.

## Simple Summary

Upper tract urothelial carcinoma (UTUC) is a rare cancer with limited epidemiological data from Central and Eastern Europe. Using nationwide Polish and European data from two decades, we analyzed UTUC incidence, mortality, and survival patterns. Poland shows a distinct sex-specific epidemiological pattern, with a smaller gap between incidence and mortality compared to most European countries. These findings highlight the need for better national registries and centralized care to improve patient outcomes.

## 1. Introduction

Upper urinary tract urothelial carcinoma (UTUC) encompasses malignancies originating from the urothelial lining of the renal calyces, pelvis, and ureter [1,2].

Established risk factors for UTUC include environmental, occupational, genetic, and iatrogenic exposures. Environmental and occupational risks include aromatic amines, diesel exhaust fumes, arsenic-contaminated water, aristolochic acid, and cigarette smoking [2,3,4]. Genetic susceptibility and blackfoot disease, a vasculitis associated with chronic arsenic exposure, are also contributors. Iatrogenic risk factors include prior treatment with cyclophosphamide or ifosfamide and long-term chronic dialysis [2,4,5]. According to the European Association of Urology (EAU) guidelines, the average latency period following risk factor exposure is approximately 20 years, with a mean exposure duration of 7 years [6].

In the European Union, UTUC is classified as a rare cancer, affecting fewer than one in two thousand individuals [7]. UTUC accounts for 5–10% of all urothelial carcinomas, with an estimated annual incidence of about two cases per hundred thousand population in Western countries [2,8,9].

Despite growing global awareness of UTUC, comprehensive epidemiological data remain limited in the Central and Eastern European region. Existing reports suggest lower incidence rates compared with Western Europe but a proportionally higher mortality burden. Furthermore, long-term population-based analyses addressing sex-specific trends, birth cohort effects, and the relationship between incidence and mortality are scarce for this region. Consequently, the epidemiological characteristics of UTUC in Central and Eastern Europe are not yet fully defined. This gap necessitates dedicated studies aimed to better understand disease distribution and outcomes across populations. Similar investigations devoted to bladder cancer revealed significant sex diversity and had profound impact on the diagnosis and treatment of the disease [10].

Therefore, the aim of this study was to analyze long-term trends in UTUC incidence, mortality, and survival in Poland using data from the Polish National Cancer Registry (PNCR) over the period 2000–2022 and to compare these findings with corresponding data from other European countries derived from the European Cancer Information System (ECIS).

Poland’s UTUC patterns may differ from other European countries due to variations in healthcare system structure, diagnostic capacity, cancer registry coverage, and population-level exposure to environmental and occupational risk factors.

In this study, age-standardized rates were calculated to facilitate accurate comparison. By providing a comprehensive, population-based analysis, this study seeks to address existing gaps in knowledge and contribute robust epidemiological evidence from Central and Eastern Europe.

## 2. Materials and Methods

Data on UTUC incidence in Poland were obtained from the Polish National Cancer Registry (PNCR) (https://onkologia.org.pl/ (accessed on 25 May 2025)), while mortality statistics and life tables were sourced from Statistics Poland (https://stat.gov.pl/en/ (accessed on 25 May 2025)). The dataset included patient sex and tumour location (renal pelvis or ureter).

There are no duplicate records in the Polish National Cancer Registry database. The data is stored in one central database, so each report is verified whether or not the case has already been reported to the register based on the patient’s national identification number. Registration takes place in accordance with ENCR guidelines.

According to Polish legislation, individual-level data from the Polish Cancer Registry can be used for statistics in aggregate form and for scientific purposes. The Polish National Cancer registry obeys strict regulations to secure confidentiality and protection of individuals. This study was conducted in accordance with the Strengthening the Reporting of Observational Studies in Epidemiology (STROBE) guidelines.

The relatively high proportion of cases with unknown stage at diagnosis was considered when interpreting the results, which nevertheless allow assessment of overall population-level patterns.

European morbidity and mortality data were collected from the European Cancer Information System (ECIS) (https://ecis.jrc.ec.europa.eu/ (accessed on 24 June 2025)).

For Poland, the analysis covered the period from 2000 to 2022, and for European countries, the data spanned 2000 to 2020. Cases were defined according to the International Classification of Diseases (ICD-10) codes C65 (renal pelvis cancer) and C66 (ureteral cancer), with carcinoma in situ and noninvasive papillary tumours excluded.

Age-standardized rates (ASRs), adjusted to the European Standard Population [11], were calculated to account for demographic differences in the study population. Temporal trends were visualized using the three-year moving average of the ASRs.

To assess the dynamics of incidence and mortality over time, the Joinpoint Regression Model version 5.2.0.0 was employed to estimate the Average Annual Percent Change (AAPC). In the Joinpoint model, a maximum of three joinpoints were set and empirical quantile methods were used.

For survival analysis, nonparametric relative survival estimates were derived using the Pohar–Perme estimator (PPE). All statistical analyses were conducted using R software 4.4.2 and the *relsurv* package (the relsurv package in R is a specialized tool designed for survival analysis, particularly focusing on relative survival) for the complete follow-up period.

## 3. Results

### 3.1. Trends in UTUC Incidence and Mortality (Poland, 2000–2022)

**Figure 1:**
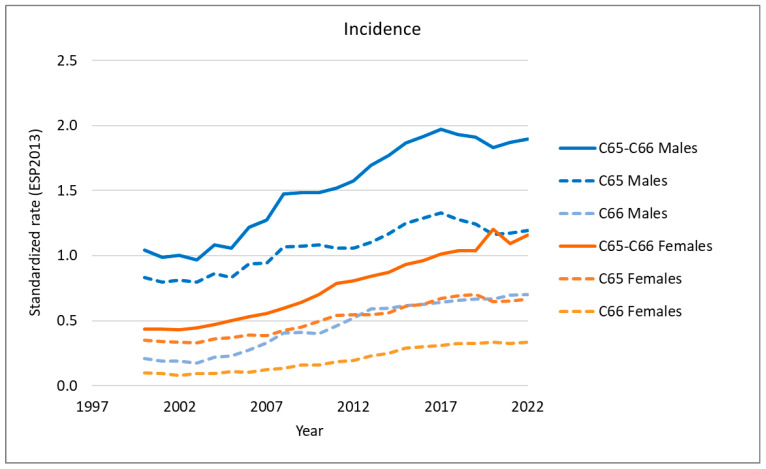
Time trends in UTUC incidence in Poland.

**Figure 2:**
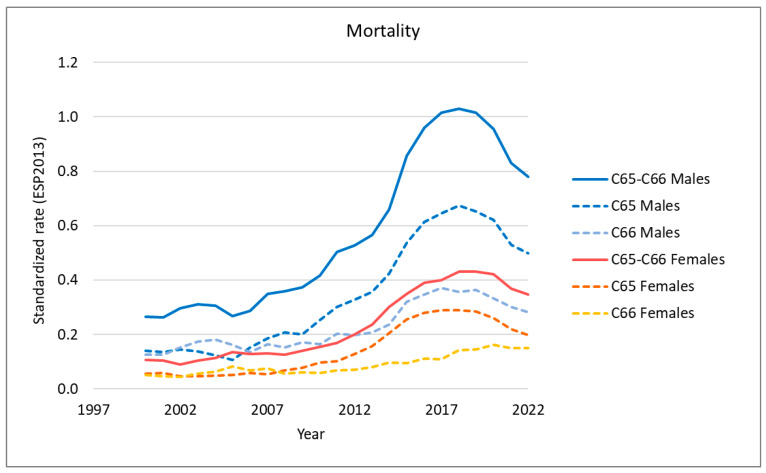
Time trends in UTUC mortality in Poland.

We analyzed the long-term trends in the incidence and mortality of renal pelvis cancer, ureteral cancer, and upper tract urothelial carcinoma (UTUC) using Polish national data (Figure 1 and Figure 2).

From 2000 to 2017, a consistent increase in both incidence (APC 5.6 in women and 9.9 in men) and mortality (APC 3.3 in women in years 2000–2009 and 16.2 in years 2009–2017; 3.5 in men in years 2000–2009 and 14.7 in years 2009–2017) was observed for UTUC in both sexes (Tables S1 and S2, Supplementary Materials).

However, during the most recent five-year period (2017–2022), a downward trend in UTUC incidence was observed in women across all types of UTUC, including renal pelvis cancer and ureteral cancer (APC −1.3). In men, this trend was reversed, with an increase in the incidence of ureteral cancer (APC 1.7) and a decrease in the incidence of renal pelvis cancer (APC −2.3).

Mortality also declined in both sexes, with a greater reduction observed in men (APC −5.47) than in women (APC −4.0). An exception occurred for ureteral cancer in women, where mortality continued to rise (APC 8.2).

It is important to highlight that both the incidence and mortality rates for renal pelvis cancer (C65) were higher than those for ureteral cancer (C66) in both sexes.

### 3.2. Comparative Analysis: Poland vs. Europe (2000–2020)

To contextualize the national data, we compared UTUC incidence and mortality trends in Poland with those of other European countries using data from ECIS for the years 2000–2020. In most European nations, including Poland, the incidence and mortality rates of UTUC have increased over the past two decades. However, some exceptions were noted. A decrease in incidence was reported in Austria and Denmark, whereas a decline in mortality was observed in Norway and the Netherlands (the latter limited to renal pelvis cancer).

**Figure 3:**
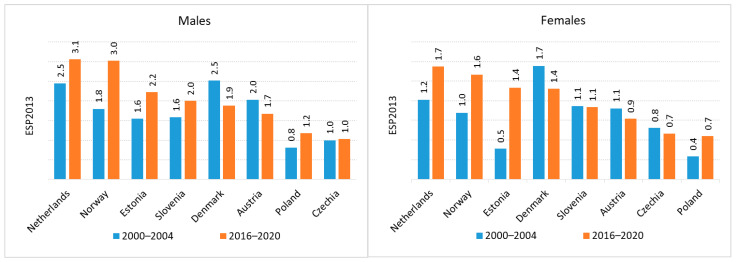
Incidence of renal pelvis cancer in Europe.

**Figure 4:**
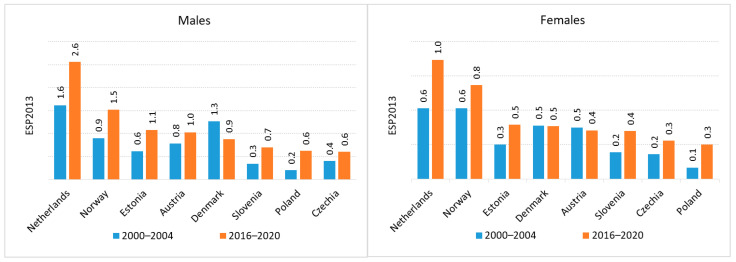
Incidence of ureteral cancer in Europe.

Among all European countries analyzed, the Netherlands exhibited the highest incidence of renal pelvis and ureteral cancers in both men and women (C65 age-standardized incidence rate (ASIR) in men 2.5 (2000–2004) and 3.1 (2016–2020), C65 ASIR 1.2 in women (2000–2004), 1.7 (2016–2020); C66 ASIR in men 1.6 (2000–2004) and 2.6 (2016–2020), C66 ASIR 0.6 in women (2000–2004), 1.2 (2016–2020). In contrast, the lowest incidences of C65 and C66 in men and women were recorded in Czechia and Poland, respectively (Figure 3 and Figure 4) (**Czechia:** C65 ASIR in men 1.0 (2000–2020), C65 ASIR 0.8 in women (2000–2004), 0.7 (2016–2020); C66 ASIR in men 0.4 (2000–2004) and 0.6 (2016–2020), C66 ASIR 0.2 in women (2000–2004), 0.3 (2016–2020); **Poland**: C65 ASIR in men 0.8 (2000–2024), 1.2 (2106–2020); C65 ASIR 0.4 in women (2000–2004), 0.7 (2016–2020); C66 ASIR in men 0.2 (2000–2004) and 0.6 (2016–2020), C66 ASIR 0.1 in women (2000–2004), 0.3 (2016–2020)).

**Figure 5:**
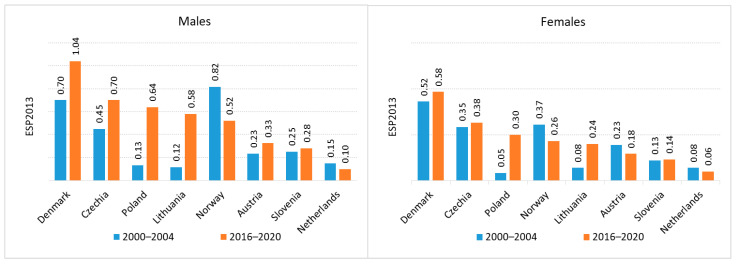
Renal pelvis cancer mortality in Europe.

**Figure 6:**
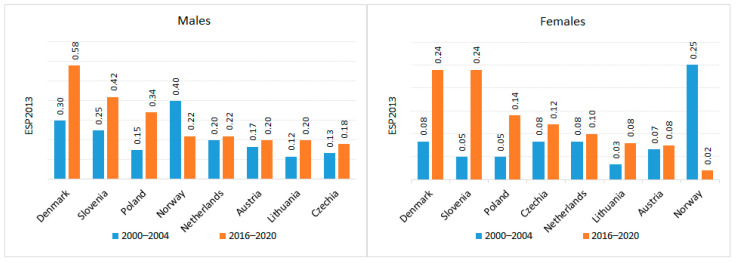
Ureteral cancer mortality in Europe.

Regarding mortality, the highest death rates for both renal pelvis and ureteral cancers were recorded in Denmark (C65 age-standardized mortality rate (ASMR) in men 0.7 (2000–2004) and 1.04 (2016–2020), C65 ASMR 0.52 in women (2000–2004), 0.58 (2016–2020); C66 ASMR in men 0.3 (2000–2004) and 0.58 (2016–2020), C66 ASMR 0.08 in women (2000–2004), 0.24 (2016–2020)). The lowest mortality rates for C65 were observed in the Netherlands for both sexes (C65 ASMR in men 0.15 (2000–2004) and 0.10 (2016–2020), C65 ASMR 0.08 in women (2000–2004), 0.06 (2016–2020); while the lowest C66 mortality rates were reported among men in Czechia (0.13 (2000–2004), 0.18 (2016–2020)) and women in Norway (0.25 (2000–2004), 0.02 (2016–2020)) (Figure 5 and Figure 6).

Poland, together with the Czechia, Denmark and Slovenia, is among the countries where the difference between incidence and mortality is smaller than in the other countries discussed.

### 3.3. Renal Pelvis Cancer (ICD-10 C65) According to Sex and Age, Poland, 2000–2022

Between 2000 and 2022, the incidence and mortality rates of renal pelvis cancer in Poland were higher in men than in women. Both indicators demonstrated a clear increase with age. The median age at diagnosis was 65 years for women and 67 years for men. The median age at death was 71 years for women and 73 years for men.

Among patients aged <40 years, 57 cases were reported across both sexes. This included two females and two males diagnosed between the ages of 15 and 19 years, and one male and one female aged 20–24 years. Notably, one case of congenital renal pelvis cancer was recorded in the registry (Figure S1, Supplementary Materials).

### 3.4. Ureteral Cancer (ICD-10 C66), According to Sex and Age, Poland, 2000–2022

Similarly to renal pelvis cancer, the incidence and mortality rates of ureteral cancer were higher in men than in women during the same timeframe. These rates increased with age. The median age at diagnosis of ureteral cancer was slightly elevated, at 72 years for women and 69 years for men. Notably, only one case was documented in a male under 20 years of age, and another case was reported in a female under 30 years of age. No cases of ureteral cancer were observed in neonates. The median age at death was 73 years for women and 75 years for men (Figure S2, Supplementary Materials).

### 3.5. Male-to-Female Ratios in Incidence and Mortality (2000–2020)

Throughout the 2000–2020 period, the UTUC incidence and mortality rates were higher in men than in women. For renal pelvis cancer, the sex disparity in both incidence and mortality progressively narrowed over time. In contrast, the male-to-female ratio for ureteral cancer incidence remained stable, whereas mortality disparities gradually declined (Figure S3, Supplementary Materials).

### 3.6. Cohort Analysis of Incidence Trends (2000–2022)

Cohort analysis revealed divergent patterns between the sexes in the incidence of renal pelvis cancer. Among men, the incidence declined across successive birth cohorts, beginning with those born after World War II, suggesting that individuals of the same age born later have a lower disease risk. Conversely, in women, the incidence increased among younger birth cohorts. This pattern mirrors observations in lung cancer and may reflect evolving exposure to environmental and lifestyle risk factors, such as tobacco use (Figure S4a,b, Supplementary Materials).

For ureteral cancer, the incidence increased with the transition to younger birth cohorts in both sexes, indicating a consistent temporal shift in the disease burden among newer generations (Figure S5a,b, Supplementary Materials).

### 3.7. Five-Year Survival Trends (2000–2019)

Five-year survival analyses of patients with UTUC were performed across four diagnostic periods: 2000–2004, 2005–2009, 2010–2014, and 2015–2019. The follow-up period was extended until 31 December 2022.

Survival analysis from 2000 to 2019 revealed gradual improvements in the five-year relative survival for both C65 and C66, with fluctuations across time periods and sexes.

The highest five-year survival rate for men with C65 (55%) was recorded in 2015–2019, whereas for women, peak survival (56%) occurred in 2010–2014.

The highest survival rates for C66 (57%) were recorded in men during 2000–2009 and in women (62%) during 2015–2019 (Figure S6, Supplementary Materials).

### 3.8. Tumour Stage Distribution (2018–2022)

An evaluation of UTUC staging based on the TNM classification from 2018 to 2022 revealed a relatively stable distribution of disease stages during this five-year period (Figure S7, Supplementary Materials). Data are collected for both sexes combined. After accounting for approximately 27% of cases with missing staging data, the analysis showed that approximately one-third of patients were diagnosed at Stage I. Stage II accounted for approximately 10–12% of cases, while Stage III included roughly one-quarter of the cohort. Stage IV diagnoses ranged from 25% to 33% of all reported cases.

### 3.9. Histological Subtypes of UTUC (2018–2022)

The histological distribution of UTUC cases in Poland from 2018 to 2022 revealed that transitional cell carcinoma was the predominant subtype, accounting for approximately 80% of the cases (Figure S8, Supplementary Materials). The remaining 20% comprised other morphological variants and malignant neoplasms not otherwise specified (NOS), indicating limited but present heterogeneity in tumour pathology. Data are collected for both sexes combined.

## 4. Discussion

Our study demonstrated significantly higher incidence and mortality rates of UTUC across Europe compared to the initial observation period, consistent with the data from Poland. One plausible contributing factor is the expanded role of retrograde intrarenal surgery (RIRS) in managing upper urinary tract malignancies, which may have improved the early detection of UTUC [12]. Additionally, heightened cancer awareness among patients and healthcare providers in Europe may have facilitated earlier diagnosis, including detection at lower tumour stages and grades, thus improving treatment outcomes.

We also confirmed that renal pelvis carcinoma occurs more frequently than ureteral carcinoma in both sexes. This finding is supported by the EAU guidelines [6] and may be explained by anatomical differences, specifically the larger surface area of the renal pelvis and prolonged exposure to urine compared to the ureter.

In our analysis, we observed a consistent male predominance in both renal pelvis and ureteral cancers, with the incidence increasing with age and over time. These observations are in line with trends reported in countries such as England [3], the Netherlands [13], Norway [14], and Spain [15].

In Poland, a more pronounced decrease in renal pelvis cancer incidence was observed in men (AAPC −2.3) than in women (AAPC −1.3). This trend may reflect sex-specific changes in smoking behaviour; while male smoking prevalence is declining, female smoking rates have remained stable or increased, contributing to the rising lung cancer incidence among women. These patterns align with the global tobacco trends. Rink et al. [16] similarly reported that cumulative tobacco exposure was more strongly associated with adverse outcomes in women with UTUC.

The more frequent occurrence of UTUC in men is explained by the protective effect of estrogens on the urothelium and the procarcinogenic effect of androgens via androgen receptors. In an analysis of 99 cases of UTUC, androgen receptor (AR) was present in approximately 20% of tumours, and AR expression was higher in ureteral cancers than in renal pelvis cancers and tended to be more present in men than in women [17]. The higher incidence of UTUC in men may also be explained by the anatomical hypothesis, according to which the presence of the prostate and more frequent subvesical obstruction lead to urinary stasis and prolonged exposure of the upper urinary tract urothelium to carcinogens; however, there are no publications that directly confirm this hypothesis.

Furthermore, our cohort analysis revealed that the incidence of renal pelvis cancer decreased among younger male cohorts (born after WWII), whereas it increased among younger female cohorts, mirroring the patterns seen in lung cancer epidemiology. This sex-divergent cohort effect warrants further investigation, particularly with respect to evolving environmental- and lifestyle-related risk factors.

Future studies should examine the potential carcinogenic effects of e-cigarette use. In a murine model, Tang et al. [18] demonstrated that exposure to electronic cigarette smoke induced lung adenocarcinoma and bladder urothelial hyperplasia, suggesting a possible link with UTUC.

From 2017 to 2022, UTUC mortality in Poland declined in both sexes. An exception occurred for ureteral cancer in women. This sex-specific divergence may be partially explained by the later stage at diagnosis in female patients than in males.

The median age at diagnoses for C65 and C66 in Poland (65–72 years) aligns with data from Spain and Sweden and is slightly lower than that in Norway and England [3,13,14,19]. Our findings also indicated a recent reduction in UTUC mortality in both sexes, particularly among men. However, ureteral cancer mortality in women has increased, potentially reflecting delayed diagnoses or disparities in access to early treatments.

Compared with other European countries, Poland has similar cancer-specific survival outcomes. For instance, the five-year UTUC survival rate in Poland is comparable to that reported in Norway, England [3,14], and Netherlands [13].

A smaller difference between incidence and mortality in Poland may suggest some underreporting of the incidence.

An evaluation of UTUC staging in Poland indicates that a considerable proportion of patients with UTUC in Poland continue to be diagnosed at an advanced stage of the disease.

No strong evidence supports the causative role of UTUC environmental risk factors, with the exception of smoking and aristolochic acid. Smoking increases the relative risk of developing UTUC by two-and-half- to seven-fold [20,21,22]. Moreover, less than 10% of individuals exposed to aristolochic acid develop UTUC [23].

Aristolochic acid has been linked not only to UTUC, but also to bladder cancer, renal cell carcinoma, hepatocellular carcinoma, and intrahepatic cholangiocarcinoma [24]. Interestingly, aristolochic acid-associated UTUC is more common in females [25,26].

Arsenic-contaminated water, the other environmental risk factor of UTUC, is especially common in Taiwan and Chile [27,28].

In addition, alcohol is also an environmental risk factor of UTUC. A large case–control study showed that compared to one who never drinks, the risk threshold for UTUC was >15 g of alcohol/day [29].

We include Lynch syndrome among the genetic risk factors for UTUC. After colorectal and endometrial cancers, UTUC is the third most common malignancy in the Lynch syndrome spectrum [30]. Patients diagnosed before the age of 60 and with a personal history of other Lynch-associated malignancies may have an increased likelihood of having Lynch syndrome [31].

### Strengths and Limitations

This study represents the first comprehensive analysis of the epidemiology of UTUC in Central Europe, including a sex-specific perspective based on data from both Polish and broader European populations. One of the principal strengths of this study lies in the use of a population-based cancer registry that encompasses the entire Polish population, ensuring high data completeness owing to active and systematic registration practices.

Importantly, this study is the first to separately evaluate renal pelvis and ureteral cancers by sex using the most recent and robust data available. Moreover, this is the first study to examine the long-term incidence trends of renal pelvis and ureteral cancer within a single country over an extended time frame (2000–2022) and to assess the five-year relative survival across a 19-year period.

However, this study has some limitations. The rarity of UTUC inherently limits the statistical power of international comparisons and trend analysis. Furthermore, the study provides a broad epidemiological overview but lacks granular clinical details, such as tumour staging at the individual level by sex, which makes interpretation of survival difficult, as well as comprehensive treatment data. Moreover, the relatively high proportion of cases with unknown stage at diagnosis was considered when interpreting the results, which nonetheless allowed assessment of overall population-level patterns.

The absence of information on therapeutic approaches limits the ability to interpret survival outcomes in the context of clinical management. An analysis of risk factors stratified by sex is also lacking.

## 5. Conclusions

This study reveals the increase in incidence and mortality rates of UTUC in most European countries, as well as the opposite trend in Poland regarding the difference between incidence and mortality compared to that in most European countries.

It also showed distinct sex-specific epidemiological patterns in UTUC.

Given the rarity of upper tract urothelial carcinoma, the development of robust, high-quality national registries is essential to improve epidemiological surveillance and guide health policies.

Centralisation of UTUC care and the adoption of standardized treatment protocols, as implemented in several Western European countries, should be considered a strategic priority.

Future research should integrate detailed clinical data on tumour staging at the individual level by sex, as well as treatment modalities and patient management pathways by sex to enhance the understanding of survival determinants and support the optimization of outcomes in patients with UTUC. Additionally, analyzing sex differences in time to diagnosis would be valuable.

## Data Availability

Data available on request from the authors.

## Supplementary Materials

The following supporting information can be downloaded at https://www.mdpi.com/article/10.3390/cancers18010126/s1; Figure S1. Incidence and mortality from renal pelvis cancer in Poland in 2020–2022 by sex and age; Figure S2. Incidence and mortality from ureteral cancer in Poland in 2020–2022 by sex and age; Figure S3. Male/female ratio for incidence and mortality of renal pelvis and ureteral cancer in Poland from 2000 to 2020; Figure S4a. Birth cohort incidence trends for renal pelvis cancer (ICD-10 C65) in Poland in 2000–2022, males; Figure S4b. Birth cohort incidence trends for renal pelvis cancer (ICD-10 C65) in Poland in 2000–2022, females; Figure S5a. Birth cohort incidence trends for ureter (ICD-10 C66) in Poland in 2000–2022 by sex; Figure S5b Birth cohort incidence trends for ureter (ICD-10 C66) in Poland in 2000–2022 by sex; Figure S6 Five-year survival rates for upper urinary tract urothelial carcinoma patients (ICD-10 C65, C66) diagnosed in Poland in 2000–2019; Figure S7 UTUC staging in Poland in years 2018–2022; Figure S8 Histological subtypes of UTUC diagnosed in Poland (2018–2022); Table S1. Incidence time trend change, UTUC (C65-C66); Table S2. Mortality time trend change, UTUC (C65-C66); Table S3. Incidence time trend change, renal pelvis (C65); Table S4. Incidence time trend change, ureter (C66); Table S5. Mortality time trend change, renal pelvis (C65); Table S6. Mortality time trend change, ureter (C66).
