# Supplementary material for: Current Knowledge on UTUC Epidemiology in Poland Compared to Europe
Source: Cancers (Basel). 2025 Dec 30;18(1):126. doi: 10.3390/cancers18010126 (PMC12784844; doi:10.3390/cancers18010126)
Supplement: Supplementary file 1 [file cancers-18-00126-s001.zip › cancers-4015180-supplementary.pdf]

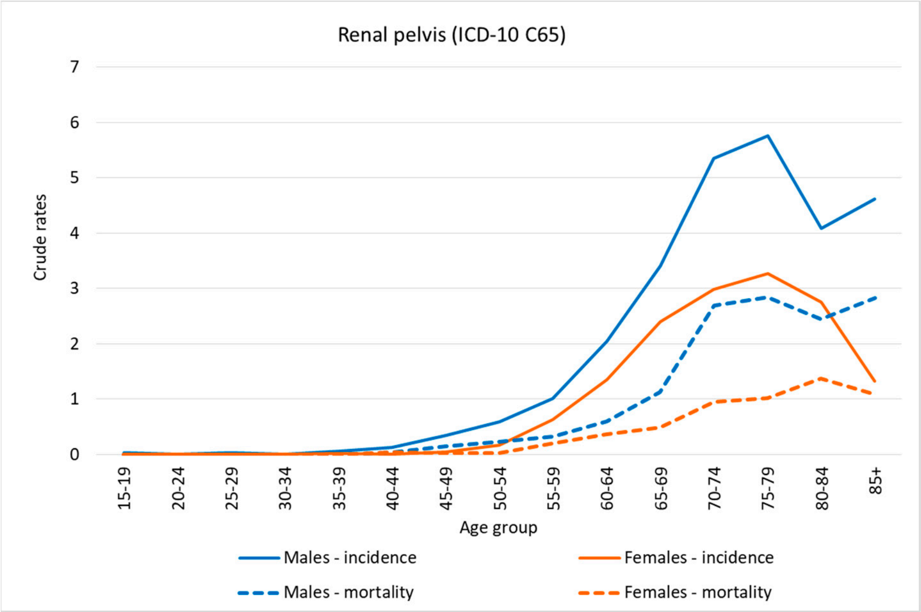

Figure S1. Incidence and mortality from renal pelvis cancer in Poland in 2020-2022 by sex and age.

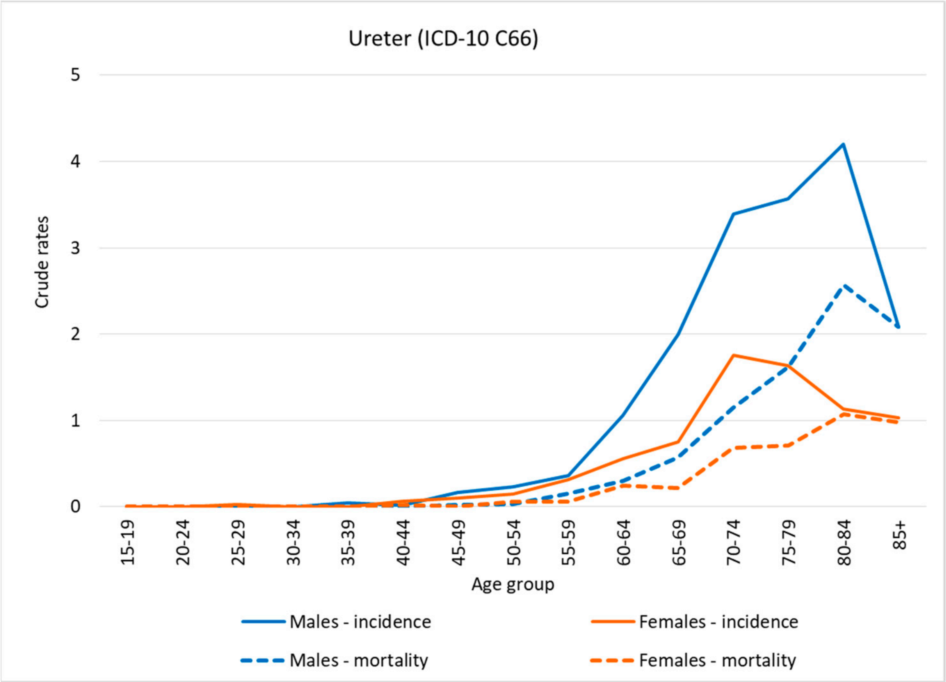

Figure S2. Incidence and mortality from ureteral cancer in Poland in 2020-2022 by sex and age.

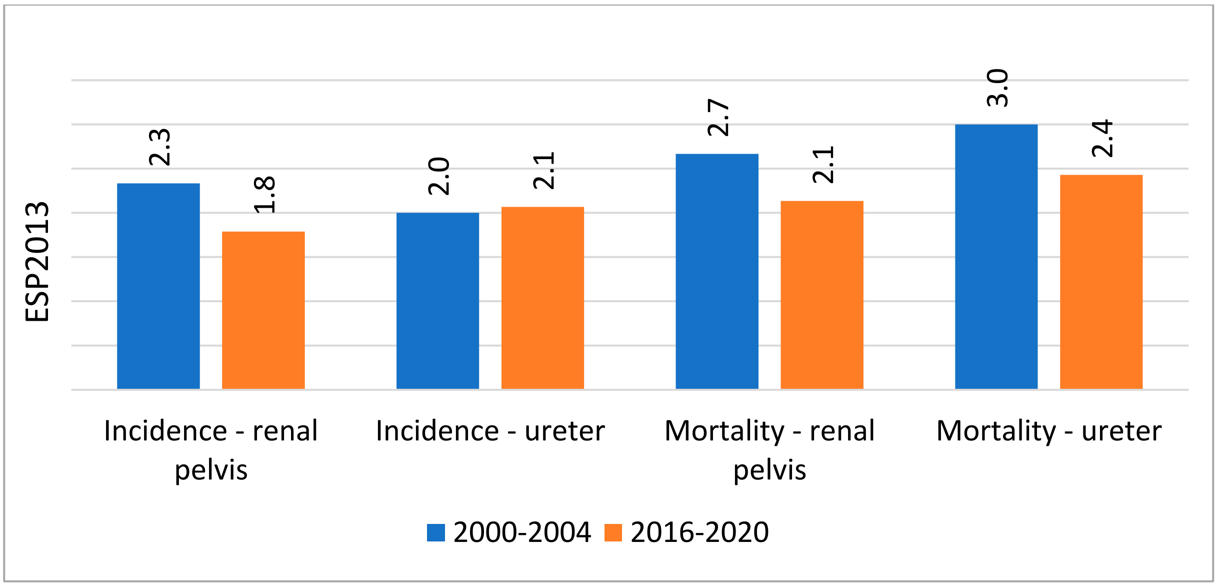

Figure S3. Male/female ratio for incidence and mortality of renal pelvis and ureteral cancer in Poland from 2000 to 2020.

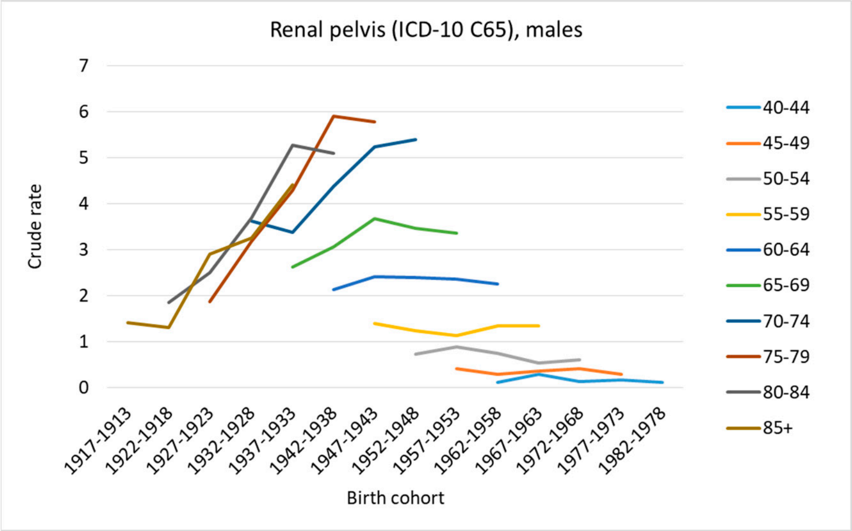

Figure S4a. Birth cohort incidence trends for renal pelvis cancer (ICD-10 C65) in Poland in 2000-2022, males.

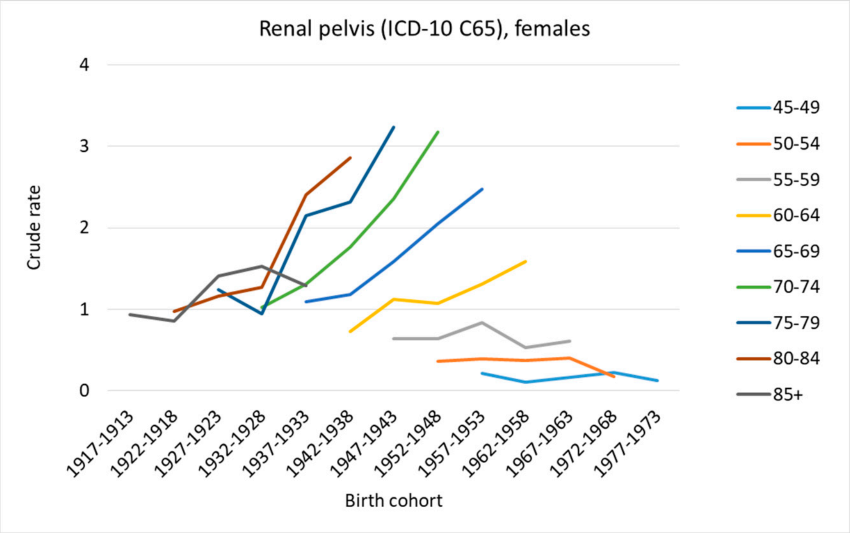

Figure S4b. Birth cohort incidence trends for renal pelvis cancer (ICD-10 C65) in Poland in 2000-2022, females.

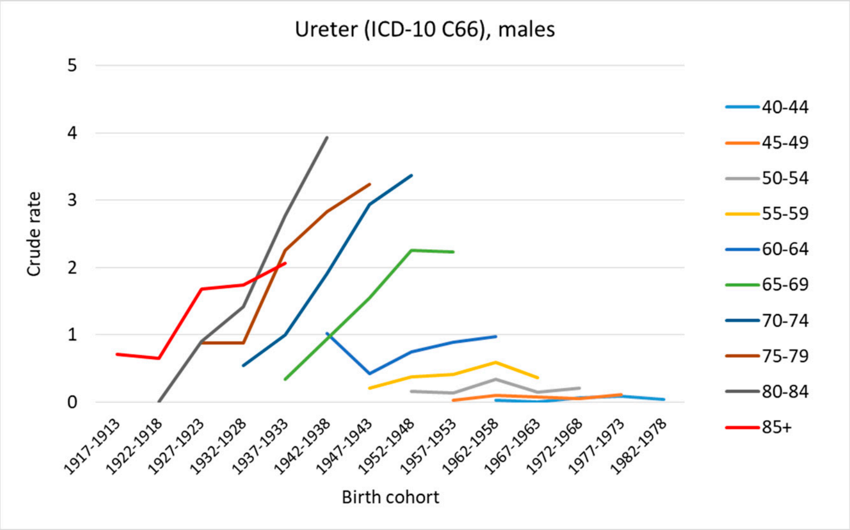

Figure S5a. Birth cohort incidence trends for ureter (ICD-10 C66) in Poland in 2000-2022 by sex.

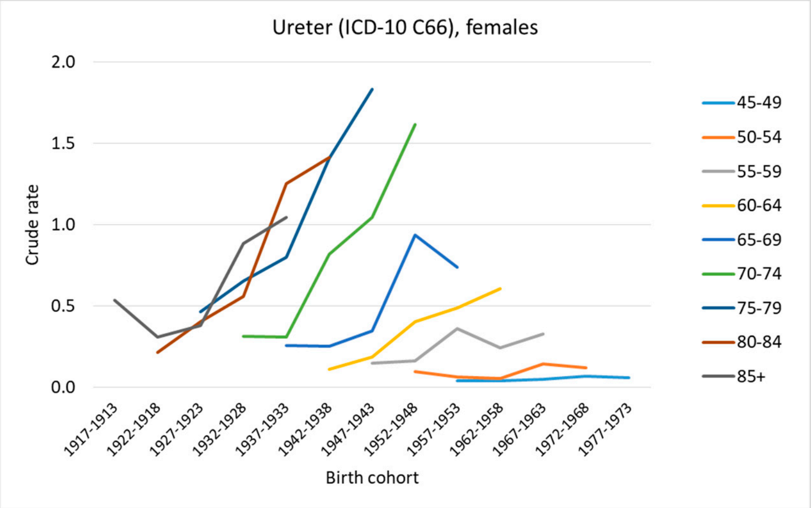

Figure S5b. Birth cohort incidence trends for ureter (ICD-10 C66) in Poland in 2000-2022 by sex.

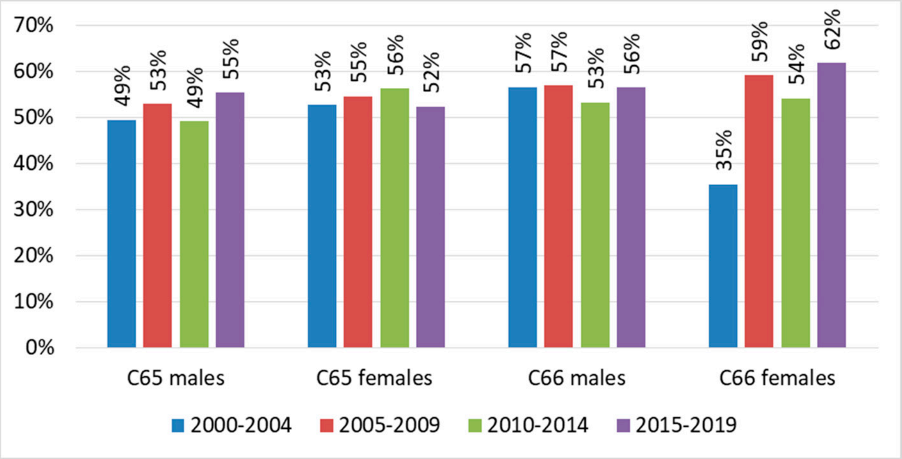

Figure S6. Five-year survival rates for upper urinary tract urothelial carcinoma patients (ICD-10 C65, C66) diagnosed in Poland in 2000-2019.

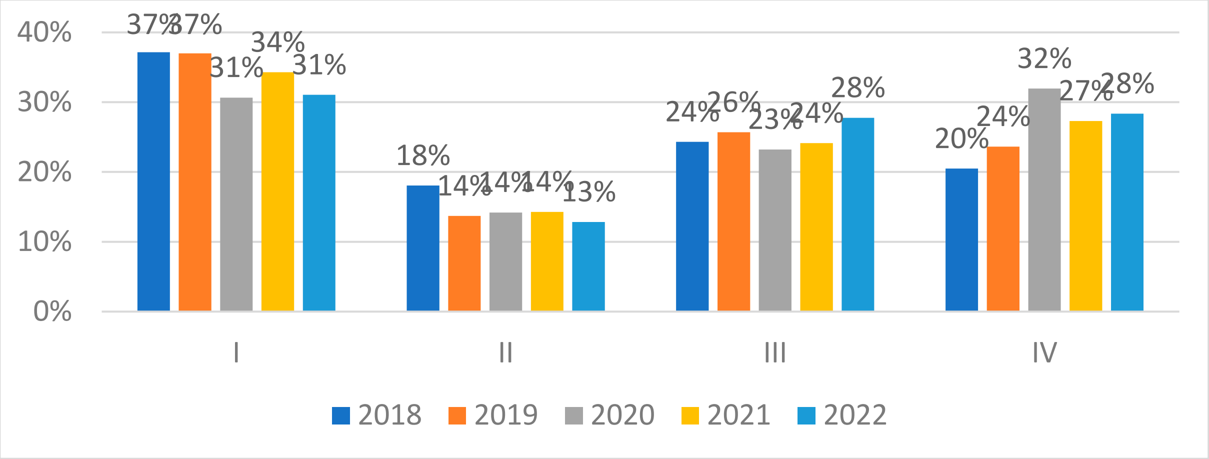

Figure S7. UTUC staging in Poland in years 2018-2022.

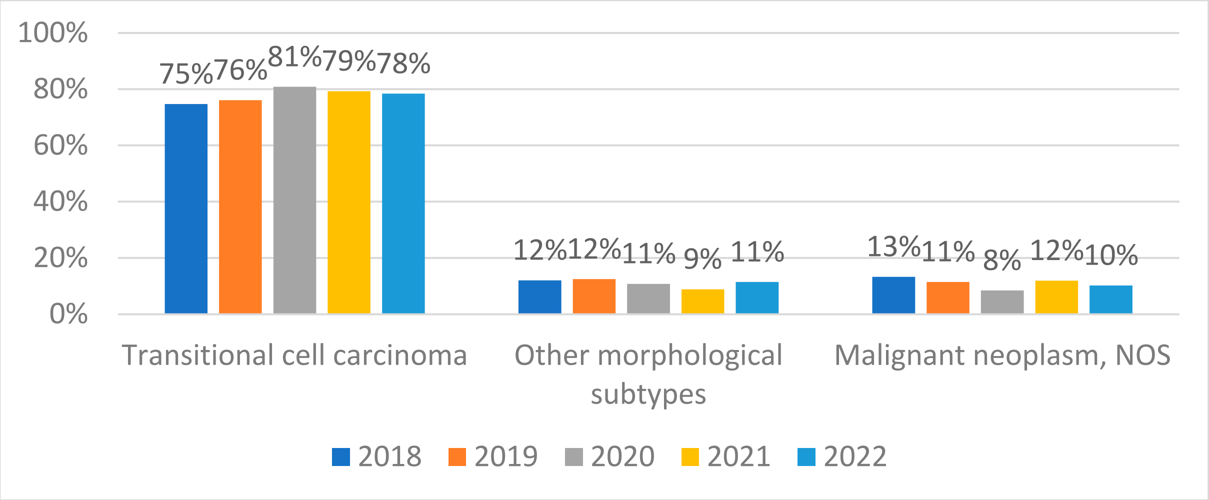

Figure S8. Histological subtypes of UTUC diagnosed in Poland (2018–2022).

Table S1. Incidence time trends change, UTUC (C65-C66).

Annual Percentage Change (APC)

Cohort	Segment	Segment Start	Segment End	APC	APC 95% LCL	APC 95% UCL	P-Value
Males	**1**	**2000**	**2014**	**9.9***	**8.5**	**12.2**	**< 0.000001**
	2	2014	2022	1.7	-4.2	4.6	0.361928
Females	**1**	**2000**	**2018**	**5.6***	**5.1**	**6.7**	**0.003999**
	2	2018	2022	-0.7	-8.6	3.8	0.704259

Table S2. Mortality time trends change, UTUC (C65-C66).

Annual Percentage Change (**APC**)

Cohort	Segment	Lower Endpoint	Upper Endpoint	APC	Lower CI	Upper CI	P-Value
Males	**1**	**2000**	**2009**	**3.5***	**1.2**	**5.2**	**0.006399**
	**2**	**2009**	**2017**	**14.7***	**12.3**	**18.9**	**< 0.000001**
	**3**	**2017**	**2022**	**-5.47***	**-9.5**	**-1.7**	**0.003599**
Females	**1**	**2000**	**2009**	**3.3***	**0.5**	**5.4**	**0.031594**
	**2**	**2009**	**2017**	**16.2***	**13.4**	**22.3**	**< 0.000001**
	3	2017	2022	-4.0	-9.1	0.4	0.075585

Table S3. Incidence time trends change, renal pelvis (C65).

Annual Percentage Change (APC)

Cohort	Segment	Segment Start	Segment End	APC	APC 95% LCL	APC 95% UCL	P-Value
Males	**1**	**2000**	**2017**	**3.1***	**2.7**	**3.7**	**< 0.000001**
	2	2017	2022	-2.3	-6.1	0.0	0.05439
Females	**1**	**2000**	**2018**	**4.5***	**4.1**	**5.5**	**0.00200**
	2	2018	2022	-1.3	-9	2.7	0.47471

Table S4. Incidence time trends change, ureter (C66).

Annual Percentage Change (APC)

Cohort	Segment	Segment Start	Segment End	APC	APC 95% LCL	APC 95% UCL	P-Value
Males	**1**	**2000**	**2014**	**9.9***	**8.5**	**12.2**	**< 0.000001**
	2	2014	2022	1.7	-4.2	4.6	0.361928
Females	**1**	**2000**	**2018**	**4.5***	**4.1**	**5.5**	**0.00200**
	2	2018	2022	-1.3	-9	2.7	0.47471

Table S5. Mortality time trends change, renal pelvis (C65).

Annual Percentage Change (**APC**)

Cohort	Segment	Lower Endpoint	Upper Endpoint	APC	Lower CI	Upper CI	P-Value
Males	1	2000	2005	-2.8	-10.3	1.8	0.19036
	**2**	**2005**	**2017**	**15.3***	**13.8**	**17.5**	**< 0.000001**
	**3**	**2017**	**2022**	**-5.4***	**-10.3**	**-1.1**	**0.0132**
Females	1	2000	2007	0.5	-3.7	3.7	0.7467
	**2**	**2007**	**2017**	**19.8***	**17.4**	**23.1**	**< 0.000001**
	**3**	**2017**	**2022**	**-9.1***	**-14**	**-4.2**	**< 0.000001**

Table S6. Mortality time trends change, ureter (C66).

Annual Percentage Change (APC)

Cohort	Segment	Lower Endpoint	Upper Endpoint	APC	Lower CI	Upper CI	P-Value
Males	1	2000	2010	1.4	-1.6	3.3	0.245951
	**2**	**2010**	**2017**	**12.7***	**9.2**	**23.5**	**< 0.000001**
	**3**	**2017**	**2022**	**-5.3***	**-11.5**	**-0.8**	**0.019596**
Females	1	2000	2011	3.0	-10.0	5.7	0.344731
	**2**	**2011**	**2022**	**8.2***	**5.5**	**22.7**	**0.021596**
